# Robust Distribution-Aware Ensemble Learning for Multi-Sensor Systems

**DOI:** 10.3390/s25030831

**Published:** 2025-01-30

**Authors:** Payman Goodarzi, Julian Schauer, Andreas Schütze

**Affiliations:** Laboratory for Measurement Technology, Saarland University, 66123 Saarbrücken, Germany; j.schauer@lmt.uni-saarland.de (J.S.); schuetze@lmt.uni-saarland.de (A.S.)

**Keywords:** prognostics and health management (PHM), sensor-based systems, AutoML, deep ensemble learning, out-of-distribution (OOD) detection, domain adaptation, structural health monitoring, condition monitoring, anomaly detection

## Abstract

Detecting distribution and domain shifts is critical in decision-sensitive applications, such as industrial monitoring systems. This paper introduces a novel, robust multi-sensor ensemble framework that integrates principles of automated machine learning (AutoML) to address the challenges of domain shifts and variability in sensor data. By leveraging diverse model architectures, hyperparameters (HPs), and decision aggregation strategies, the proposed framework enhances adaptability to unnoticed distribution shifts. The method effectively handles tasks with various data properties, such as the number of sensors, data length, and information domains. Additionally, the integration of HP optimization and model selection significantly reduces the training cost of ensemble models. Extensive evaluations on five publicly available datasets demonstrate the effectiveness of the proposed framework in both targeted supervised tasks and unsupervised distribution shift detection. The proposed method significantly improves common evaluation metrics compared to single-model baselines. Across the selected datasets, the framework achieves near-perfect test accuracy for classification tasks, leveraging the AutoML approach. Additionally, it effectively identifies distribution shifts in the same scenarios, with an average AUROC of 90% and an FPR95 of 20%. This study represents a practical step toward a distribution-aware front-end approach for addressing challenges in industrial applications under real-world scenarios using AutoML, highlighting the novelty of the method.

## 1. Introduction

There is a general assumption in supervised machine learning (ML) that the training and test data come from the same distribution, referred to as in-distribution (ID) [1]. However, in real-world scenarios, data distributions often deviate from the training distribution due to various reasons, such as changes in operational conditions, sensor degradation, or environmental variations [2,3,4]. This phenomenon, known as a distribution shift or domain shift, can result in out-of-distribution (OOD) data (inputs that deviate from the distribution observed during training) and lead to reduced accuracy of trained models [5,6]. This issue is particularly challenging in safety-critical applications and in industrial condition monitoring, where it may cause inaccurate predictions or undetected failures [7,8,9]. Monitoring deployed models is crucial to ensure reliable and robust condition monitoring, as it helps identify when a model requires maintenance, e.g., updating or expanding [10,11,12].

Various methods exist to address distribution shifts during testing, including transfer learning [13], domain adaptation [14], and domain generalization [15,16] techniques. However, none of these methods can fully guarantee the prevention of failures in deployed systems [15]. In condition monitoring, the risk of failure is particularly high due to the typically limited number of independent observations, the time-consuming nature of data acquisition, and the difficulty in covering all possible operational conditions [3]. Therefore, detecting potential OOD inputs is essential to improve the reliability and robustness of these systems [17,18].

Methods for detecting OOD have been extensively studied, leading to the development of various approaches [19]. These methods can be applied in a post-hoc manner, both unsupervised [20,21] and supervised [22,23]. Supervised methods assume the availability of some data from the shifted distribution, allowing the model to learn the characteristics of possible shifts. In contrast, unsupervised approaches are more suitable when obtaining samples from the shifted distribution is challenging or impractical. This is often the case in condition monitoring [3], where reproducing faults under new conditions is not feasible.

OOD detection methodologies can be categorized into several approaches [19]. Classification based methods use the model’s output to differentiate between ID and OOD samples. A simple OOD baseline in this category is the maximum softmax probability (MSP) method, which identifies OOD data based on the model’s highest softmax output [20]. An early extension of this approach is ODIN [21], which applies temperature scaling to improve OOD detection by adjusting the neural network’s output. The energy-based method (referred to as Energy in the rest of the article) further refines this concept by using an energy score instead of a softmax-based score [24]. Another group of methods, known as density-based methods, assumes that ID data is more likely to be located in areas of high density within the feature space compared to OOD data. Techniques like the Histogram-Based Outlier Score (HBOS) [25] fall into this category, where density estimation is employed to identify anomalies.

Distance-based methods offer a different approach, relying on the idea that the embedded features of OOD samples should be relatively far from those of ID data. These methods compute the distance between input samples and the training data in the feature space to determine if the input is OOD [22,26]. Another category is OOD detection using Deep Ensembles [27,28,29], which leverages the intuition that disagreement or variation in the output of multiple models within an ensemble can effectively indicate OOD data.

Several studies address the problem using open-set classification approaches, where models are tested under conditions where a portion of faults is only introduced during the test phase. For instance, Li et al. [30] integrated stochastic elements to improve fault detection in high-speed trains, ensuring better sensitivity to previously unseen faults. In another approach, Zhou et al. [31] employed a Bayesian convolutional neural network to differentiate between known and unknown faults in a bearing dataset. Similarly, Wu et al. [32] employed Bayesian deep learning to identify unexpected faults in high-speed train bogies, focusing on capturing the uncertainty to enhance differentiation between known and unknown faults. Kamranfar et al. [33] proposed an anomaly detection framework using Multiple Instance Learning tailored for real-world sequential datasets. In their study utilizing a bearing dataset, they defined the damaged states of the bearings as anomalies, while the normal bearing conditions served as the non-anomalous class.

While these studies have made significant progress, the majority are limited to single use-case designs and fail to offer generalizable solutions for realistic multi-sensor applications. To address these gaps, this paper makes the following key contributions:

- We propose a multi-sensor deep ensemble framework that improves fault detection accuracy while effectively identifying domain shift and distribution shift.
- We integrate hyperparameter (HP) optimization into model training to generalize the ensemble framework for various condition monitoring and fault detection tasks.
- The proposed method is evaluated on multiple industrial condition monitoring datasets, demonstrating its generalizability and robustness in real-world scenarios.

The remainder of this paper is organized as follows: Section 2 reviews the background and related works. Section 3 describes the datasets used. Section 4 explains the proposed methodology, and Section 5 presents the results, followed by a discussion in Section 6. Finally, Section 7 concludes the paper and outlines future research directions.

## 2. Background and Related Works

### 2.1. Hyperparameter Optimization

Modern L models have many parameters optimized during training and HPs that are fixed during training but need to be optimized for each task. Hyperparameter optimization (HPO) is one of the tasks in automated ML (AutoML) [34,35] that focuses on automatically finding the optimal HPs for a given task.

For example, in deep neural networks (DNNs), weights and biases are optimized during training. However, the optimization process itself relies on several HPs that significantly influence the final performance of the trained network. These training HPs include the learning rate, learning rate schedule, regularization method, and the training optimizer.

In addition to training HPs, defining a network architecture involves numerous design decisions. For example, in convolutional neural networks (ConvNets), important architectural HPs include the number of convolutional layers, kernel size, and stride size. Neural Architecture Search (NAS) is a branch of AutoML dedicated to optimizing these architectural HPs and, more broadly, identifying the best architecture for a given task within a defined search space. The search space encompasses all valid combinations of HPs for the task.

Various NAS algorithms have been proposed, differing in how they define the search space and implement search strategies. Incorporating expert knowledge can help constrain the search space to a more efficient subset of HPs, significantly reducing computational cost while maintaining performance.

Grid search and random search are baseline strategies for HPO. However, both are particularly computationally expensive for NAS applications. Bayesian optimization [36] is a state-of-the-art strategy for global optimization of expensive black-box functions. It consists of two main components: a surrogate model and an acquisition function. After each trial, the surrogate model is updated based on the available observations, and the acquisition function evaluates the performance of candidate points. In practice, the number of iterations is often limited by the computational resources allocated to the task.

### 2.2. Model Ensembles for Robust Predictions

Ensemble methods enhance the prediction accuracy of ML systems [27]. By combining predictions from multiple models, ensembles create a more robust and powerful predictive framework. There are several approaches to building ensemble models, including bagging, boosting, and stacking. Stacking, in particular, offers flexibility by allowing independent training of base models and aggregating predictions from different types of models.

Deep ensembles, which involve multiple neural networks, have demonstrated superior performance across a wide range of applications, providing greater reliability and accuracy compared to individual models [28]. Various strategies have been proposed to increase diversity within deep ensembles, as diversity is a key factor in improving performance. One early approach involved training ensembles by initializing networks with different random seeds, resulting in diverse sets of weights across models [28]. Additionally, introducing diversity through variations in training batches and data selection has been shown to enhance results [37]. Further diversity can be achieved by employing different HPs and model architectures, which boosts the performance of ensembles [38,39].

Predictions from aggregated results can be made using various strategies, such as majority voting, averaging, or weighted combination methods. When the ensemble comprises diverse models with potentially complementary strengths, a meta-classifier can be introduced to learn how to best combine their outputs [40]. The use of a meta-classifier adds flexibility and can improve performance by leveraging patterns in the predictions of the base models. The meta-classifier can range from a simple logistic regression model to a more complex method, such as gradient boosting [41]. The choice of the meta-classifier depends on factors such as the dataset’s size, the base models’ diversity, and computational constraints.

### 2.3. Out-of-Distribution Detection

In this study, alongside our proposed ensemble approach, we evaluated several alternative OOD detection techniques that are based on individual neural networks.

**Maximum Softmax Probability (MSP)** ref. [20] is a widely used indicator for OOD detection. This method is simple to apply since it relies on the standard neural network output without modifying the training process. The score is computed as follows:

(1) $$\mathrm{MSP}=\max_{i}\left(\frac{e^{z_{i}}}{\sum_{j}e^{z_{j}}}\right)$$

where $z_{i}$ represents the logit (input to the softmax function) for class *i*.

**ODIN** ref. [21] is an early method that improves OOD detection using softmax scores by applying temperature scaling and input perturbation. It aims to enhance the separation between ID and OOD samples. The ODIN score is calculated as follows:

(2) $$\mathrm{ODIN}=\max_{i}\left(\frac{e^{z_{i}/T}}{\sum_{j}e^{z_{j}/T}}\right)$$

where *T* is the temperature scaling parameter.

**Energy** ref. [24] computes an energy score to measure the likelihood that a sample belongs to the ID data, with lower scores typically indicating ID samples and higher scores indicating OOD samples. The energy score is given by

(3) $$E=-T\cdot \log\left(\sum_{i}e^{z_{i}/T}\right)$$

The **Histogram-Based Outlier Score (HBOS)** ref. [25] is a density-based method that uses the distribution of feature values to detect outliers. The method relies on creating histograms for each feature, where the frequency of the data points in each bin is used to compute the outlier score.

**Mahalanobis distance-based OOD detection (MDS)** ref. [42] uses the features extracted from the penultimate layer of a neural network to determine if a given sample is OOD. Instead of calculating the distance to a single mean, the Mahalanobis distance is computed for each class, and the minimum distance across all classes is used as the OOD score.

MDS(x) for a sample *x* is given by

(4) $$MDS\left(x\right)=\min_{i}\sqrt{{\left(f\left(x\right)-\mu_{i}\right)}^{T}\Sigma^{-1}\left(f\left(x\right)-\mu_{i}\right)}$$

where

- f(x) is the feature vector of the input sample *x* from the penultimate layer;
- $\mu_{i}$ is the mean vector of the ID features for class *i* (computed during training);
- $\Sigma^{-1}$ is the inverse of the shared covariance matrix of the ID features across all classes.

**OOD Detection Using Statistical Moments of Data (StatData)** is a method that relies on simple features extracted from raw data. In certain use cases, distribution shifts can be identified directly from the input data. To evaluate this approach in our benchmark, we construct a *k*-nearest neighbor [43] (kNN) model based on the statistical moments of the input data, including mean, variance, skewness, and kurtosis, as described in Algorithm 1. This method leverages the inherent distributional properties of sensor data to classify samples as either ID or OOD.
**Algorithm 1** OOD Detection Using Statistical Moments of Data  1:**Input:** *S* sensors, in-distribution (ID) training data  2:**Output:** OOD score for new samples  3:**for** each sensor s=1 to *S* **do**  4:     Compute the statistical moments of the signal  5:**end for**  6:**features** = concatenate statistical moments across all sensors  7:Build a kNN model (k = 1) using these features on the ID data  8:**Return:** OOD score from the kNN model

### 2.4. Evaluation Metrics

Three metrics are used to evaluate the proposed methods and their results. The first metric, accuracy, is used for assessing supervised classification methods, while the other two metrics address the performance of unsupervised methods.

**Accuracy**: To evaluate the prediction performance of models in supervised classification tasks, one of the essential metrics is accuracy. In tasks with approximately equal representation of classes, accuracy is one of the most useful and easy-to-interpret metrics [44]. Accuracy is defined as follows:

(5) $$\mathrm{Accuracy}=\frac{TP+TN}{TP+TN+FP+FN}$$

where:

- TP = True Positives
- TN = True Negatives
- FP = False Positives
- FN = False Negatives

**AUROC**: Area Under the Receiver Operating Characteristic curve (AUROC) shows the relation between TP rate (TPR) and FP rate (FPR) and is a threshold invariant metric in binary classification tasks [45]. A perfect model has 100% AUROC.

**FPR95**: AUROC alone cannot capture all aspects of the model’s behavior, particularly in handling false positives. FPR at TPR*x* (FPR*x*) measures the likelihood of misclassifying an OOD sample as an ID sample when the TPR is fixed at *x*%. While the choice of *x* can vary depending on the task requirements, 95% (FPR95) is a commonly used standard in related studies [19]. AUROC and FPR95 should be evaluated together to gain a comprehensive understanding of the model’s performance.

## 3. The Proposed Framework

**Figure 1:**
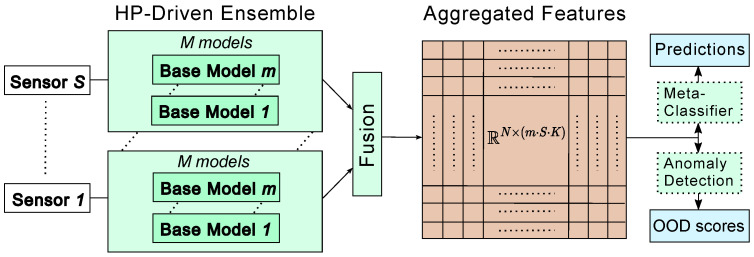
Multi-sensor ensemble framework. The method consists of four building blocks: HP-driven Ensemble, fusion, meta-classifier, and anomaly detection, with the HP-driven ensemble serving as the core component.

This section outlines the proposed framework designed to address diverse fault detection applications in real-world scenarios. Datasets from condition monitoring applications vary in data length and the number of sensors. The proposed framework is designed to accommodate these differences. As illustrated in Figure 1, the method is an ensemble-based approach that incorporates embedded HPO and provides OD scores for predictions. This approach aligns with the principles of AutoML by integrating HPO and model selection as essential components of the ML process [34,35]. Rather than being tailored to a specific use case, the framework is designed to ensure broad applicability across datasets with similar characteristics.

The framework consists of four modular building blocks: the HP-driven ensemble, fusion, meta-classifier, and anomaly detection components. Each block offers flexibility in selecting methods to suit specific requirements and constraints. Common considerations influencing these choices include hardware limitations, interpretability, and prediction performance. For instance, interpretability constraints may restrict the selection of methods for the ensemble block. In this study, we demonstrate the framework using a single configuration for each block, as summarized in Table 1. However, alternative configurations can also be employed based on specific requirements. The following sections provide a detailed description of the framework and its components.

All methods and algorithms of this study were implemented in MATLAB [46]. The DNNs were developed using the MATLAB Deep Learning Toolbox [47], while Bayesian optimization was conducted using the Statistics and ML Toolbox [48]. The training of ConvNets was performed on NVIDIA Quadro RTX 5000 GPUs to ensure computational efficiency.

### 3.1. HP-Driven Ensemble

The HP-driven ensemble block consists of multiple base models selected from the HP search space after the HPO process. Using the flexibility of the stacking approach, the selection of base models can be customized according to specific design criteria, such as deployment constraints, computational budget, interpretability requirements, or expert domain knowledge [49]. Possible options for the base models include interpretable methods [50], DNNs [28], or custom feature engineering approaches.

**Figure 2:**
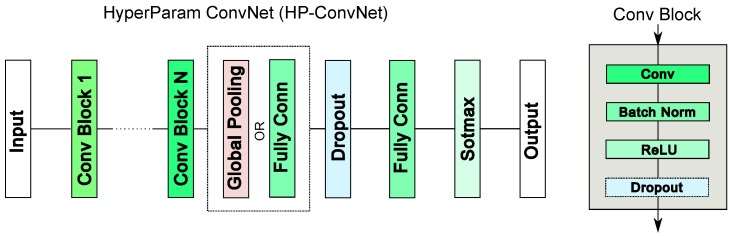
Parametric ConvNet architecture with up to *N* convolutional (conv) blocks. The corresponding HPs are listed in Table A1.

This study employs DNNs as the base models for the ensemble block. The architecture designs for DNNs vary widely, ranging from multi-layer perceptrons to ConvNets [51] and transformers [52]. ConvNets have become a cornerstone of ML applications, initially revolutionizing the field of computer vision [53] and subsequently being adapted to a wide range of domains, including signal processing, natural language processing, and more [51]. Over the years, many variations of ConvNets have been proposed [54,55,56,57]. We utilize the HyperParam Convolutional Neural Network (HP-ConvNet), a 1D-ConvNet [58], as depicted in Figure 2. HP-ConvNet is an adaptive architecture designed to accommodate a wide range of signals across diverse use cases. At its core, the network employs a convolutional (Conv) block, which integrates a convolutional layer, batch normalization, ReLU activation, and a dropout layer. A comprehensive description of HP-ConvNet, including its design and parameters, is provided in Appendix B.

The HPs of the HP-ConvNet are selected using Bayesian optimization over a defined search space, as summarized in Table A1. This study primarily focuses on architecture HPs, while the training HPs, except for the learning rate (LR), remain fixed. The training optimizer for all evaluations is Adam optimization [59]. Given the large number of potential HPs for the HP-ConvNet, numerous valid network configurations are possible [60]. To reduce the search space and facilitate early rejection of invalid HP combinations, several constraints are applied to the optimization algorithm, as detailed in Appendix C.

### 3.2. Fusion

The scores or embedded features generated by the ensemble block must be aggregated to create a unified representation suitable for subsequent processing. The data fusion process can range from simple concatenation to more complex operations, such as weighted averaging or advanced transformation techniques [61]. Aggregating features from different models may require additional preprocessing steps, including dimensionality reduction or feature selection, to ensure consistency and reduce redundancy.

In this study, we assign equal weights to each base model within the ensemble block, with decision-level fusion employed as the integration point. The ensemble block outputs softmax scores generated by each ConvNet, where all scores share the same dimensions, corresponding to the number of classes in the labels. The aggregated matrix, formed by concatenating the softmax scores, is represented as m×S×K, where *S* is the number of sensors, *m* the number of selected models per sensor, and *K* is the number of classes in the classification task.

### 3.3. Ensemble Prediction

The primary output of the workflow consists of the predictions for the defined task, which are computed using the aggregated features. Following the stacking approach, a meta-classifier is used to generate the final prediction by combining the outputs of the ensemble block. In this study, as reported in Table 1, we utilize adaptive boosting (AdaBoost) [62] as the meta-classifier. AdaBoost is an iterative ensemble method that combines a series of weak learners, typically decision trees, to form a strong classifier. The selection of AdaBoost as the meta-classifier is motivated by its ability to handle diverse input distributions and its effectiveness with smaller datasets [63].

### 3.4. Ensemble-Based OOD Detection

Ensemble-based OOD detection leverages the intuition that disagreement or variation among the outputs of multiple models can effectively signal the presence of OOD data. This method involves training an ensemble of models, often neural networks with different initialization [28], HPs [39], or architectures [38], and combining their predictions to detect OOD samples.

In the proposed framework, we employ a non-parametric anomaly detection approach on the aggregated scores generated by the ensemble to compute the OOD detection scores. As outlined in Table 1, we employ k-Nearest Neighbors (kNN) as the anomaly detection method, inspired by its effectiveness in prior studies [64]. kNN is a straightforward, distance-based approach that provides interpretable results by quantifying the similarity between a new sample’s aggregated features and those of the ID data. This method is particularly appealing due to its simplicity and ability to adapt to different distributions without requiring extensive parameter tuning. However, alternative anomaly detection techniques can also be utilized within the framework, depending on specific requirements. Examples include one-class SVM, isolation forests, or variance-based thresholds on aggregated features. Appendix D provides a comparison of four different possible methods for anomaly detection within this framework.

### 3.5. Training Multi-Sensor Deep Ensemble

Using the selected methods outlined in Table 1, we integrate diverse HPs within the multi-sensor framework to construct the deep ensemble model. The training process, detailed in Algorithm 2, utilizes an HP search budget of M=100 trials. HPO of the base model (HP-ConvNet) is conducted for each sensor *M* times using the defined search space. Based on validation performance, the top *m* models are selected from the *M* trained models. In total, m×S base models are selected, and their softmax scores are concatenated to form the aggregated features.

The workflow described is based on the selected methods from Table 1; however, the approach remains similar for other possible configurations. The framework’s model-specific element lies in selecting the base models for the HP-driven ensemble and defining the corresponding HP search space. Notably, due to the stacking-based ensemble block, a mixture of different ML methods or DNN architectures can be utilized, depending on the properties of the input signals.

The meta-classifier and anomaly detection methods are trained using the aggregated features. During inference, the trained deep ensemble model, meta-classifier, and kNN model are employed to predict class labels and compute OOD scores for each new input.

Selecting an appropriate data planning strategy within this framework is critical to avoiding overfitting and data leakage. Specifically, ID-test data must remain excluded from the HPO and training processes. In this study, the data is divided into 50% training, 20% validation, and 30% test sets. For small datasets, cross-validation is also a viable approach to ensure robust evaluation.
**Algorithm 2** Multi-Sensor Deep Ensemble Training  1:**Input:** *S* sensors, *M* trials for HP search per sensor  2:**Output:** Trained deep ensemble model, meta-classifier, and kNN model  3:**for** each sensor s=1 to *S* **do**  4:     Perform HP search using HP-ConvNet with *M* trials for sensor *s*  5:     Select the top *m* networks based on validation performance from the *M* trials  6:**end for**  7:Concatenate the softmax scores of all selected models across sensors  8:Train the meta-classifier on the aggregated softmax scores for final prediction  9:Train the kNN model on the aggregated softmax scores for OOD detection10:**Return:** Trained deep ensemble model, meta-classifier, and kNN model

## 4. Datasets

Five publicly available datasets from industrial condition monitoring and fault detection were used. This section describes the datasets used in the study.

- The ZeMA Electromechanical Axis (EA) dataset [65];
- The ZeMA Hydraulic System (HS) dataset [66];
- The Open Guided Waves (OGW) dataset [67];
- The Paderborn University Bearing (PU) dataset [68];
- The Case Western Reserve University Bearings (CWRU) dataset [69].

The aim was to select datasets with multiple sensors and multiple domains. Many industrial datasets consist of readings from multiple sensors. These recordings may capture different physical properties, such as vibration, pressure, or current, across one or more components. Vibration, in particular, is a property that can be measured in a wide range of applications and from multiple locations within a system (see Table 2).

In this study, a “sensor” is not limited to a physically separate device; it may also refer to a transformation or subdivision of an original recording. For example, Fourier or Wavelet transformations of vibration recordings can be considered separate sensors. Furthermore, different phases of a physical process may be treated as different sensors. Thus, sensors in this study are not restricted to concurrent or synchronized recordings.

Variations in force level, pressure, or temperature can be potential causes of shifts in the data distribution and can be treated as different domains. The datasets used in this study were not explicitly designed to address domain shift. This selection was made for two main reasons. First, there are no well-known datasets specifically designed for this purpose in the targeted field. Second, using frequently studied datasets highlights the presence of domain shift problems in real-world scenarios, underscoring the relevance and practicality of addressing these challenges.

To ensure comparability across datasets, we used balanced versions in this study. For datasets that were originally imbalanced, we applied downsampling to the majority class to create balanced versions. Table 2 provides details for each dataset, including the ranges of signal lengths, number of classes, defined domains, and sensors. Dataset descriptions covering sensor types and defined domains are provided in Appendix A.

## 5. Results

**Figure 3:**
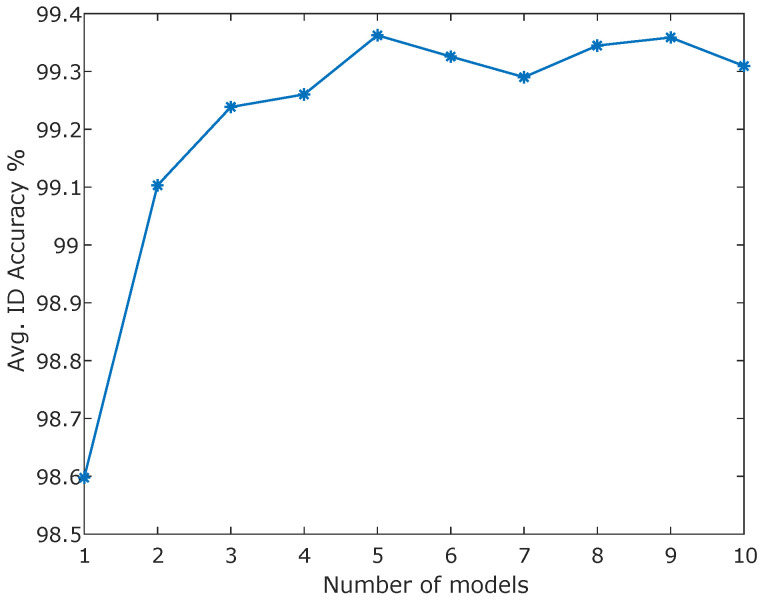
Average ID accuracies for different numbers of models.

We trained ensemble models for each use case listed in Table 2, treating all datasets as multivariate classification tasks using Algorithm 2. To maximize the model’s performance, all available sensor data were included in the modeling process for each dataset. Following the training of the HP-driven ensemble, *m* models for each sensor were selected from the pool of all trained models. The number of models in the deep ensemble, *m*, is a critical HP affecting both prediction accuracy and OOD detection efficacy. Increasing *m* generally enhances the ensemble’s generalization and anomaly-detection capabilities. While fine-tuning *m* for each dataset individually would be the preferred approach for addressing a specific task, we opted to set *m* based on the average ID accuracy across all datasets to demonstrate the generalizability of the approach. Figure 3 shows that the ID accuracy plateaued after five models, so we fixed m=5 for the remaining experiments to balance performance and computational efficiency.

**Figure 4:**
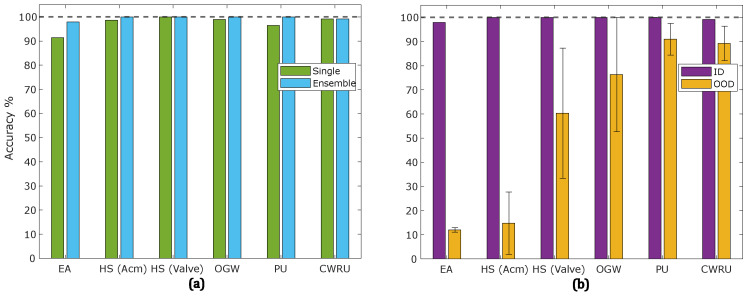
Prediction accuracy for each dataset: (**a**) ID accuracy of the best single model vs. ensemble of five models; (**b**) accuracy of the ensemble for ID and OOD data. Error bars in (**b**) show the accuracy range across different domains.

The prediction results of the models for each dataset are shown in Figure 4. Figure 4a compares the ID accuracy of the best single model with the ensemble of five models for each sensor. The accuracy improved across all datasets when using the deep ensemble. Figure 4b illustrates the accuracy of the deep ensemble model for both ID and OOD data. A clear accuracy drop from ID to OOD data is observed for all datasets, with the most significant decreases occurring in the EA and HS (Acm) datasets. The error bars in Figure 4b indicate the range between the maximum and minimum accuracy of the ensemble model across different domains. There is significant variation in the prediction accuracy of the ensemble models for most datasets. For instance, in the OGW dataset, the OOD accuracy varies between almost 50% and 100%, depending on the target domain.

**Figure 5:**
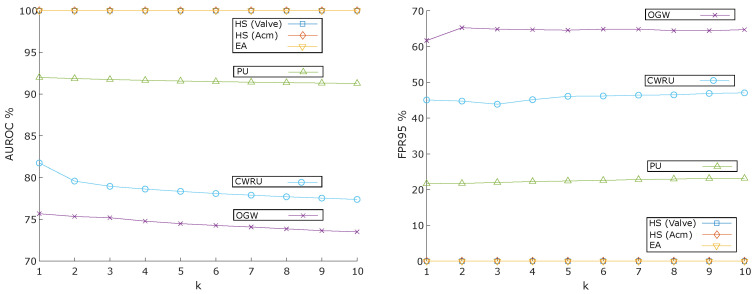
AUROC and FPR95 results across datasets as a function of varying *k* values.

As outlined in Section 3.4, the multi-sensor ensemble-based OOD detection method (Ens+kNN) employs kNN to generate OOD scores. The value of *k* is an important HP for kNN, and we ran the algorithm with *k* values ranging from 1 to 10 to examine its impact. Figure 5 presents the AUROC and FPR95 results across datasets as a function of different *k* values. For three datasets—EA, HS (Valve), and HS (Acm)—the value of *k* shows minimal effect on performance. However, increasing *k* results in a decreased AUROC for the other datasets. Consequently, k=1 was chosen for the remainder of the experiments, this is inline with the explanation from the original finding [43]. Performance metrics for other anomaly detection methods are compared in Appendix D.

The AUROC and FPR95 metrics are measured for each OOD detection method described in Section 2.3 and Section 3.4 across all datasets. Table 3 presents performance results for each method. For methods MSP, ODIN, Energy, HBOS, and MDS, we report results from the best-trained model with the highest ID validation accuracy. The Ens+kNN method consistently achieves the highest performance for both AUROC and FPR95 metrics, followed by StatData, which relies solely on input features. However, the other methods display relatively poor performance in FPR95, underscoring the superior reliability of Ens+kNN for OOD detection.

**Figure 6:**
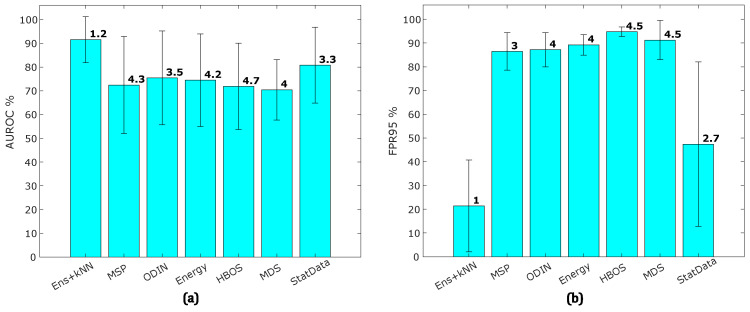
The results of the OOD detection methods: (**a**) the average AUROC and (**b**) the average FPR95 for each method. Error bars represent standard deviations across datasets.

To visualize the results more effectively, Figure 6 shows the average AUROC and FPR95 across all datasets. The error bars represent the standard deviation for each method, and the number above each bar indicates the method’s average rank across datasets. Ens+kNN consistently ranks highest in both AUROC and FPR95, with Data ranking second but exhibiting high variability in FPR95.

**Figure 7:**
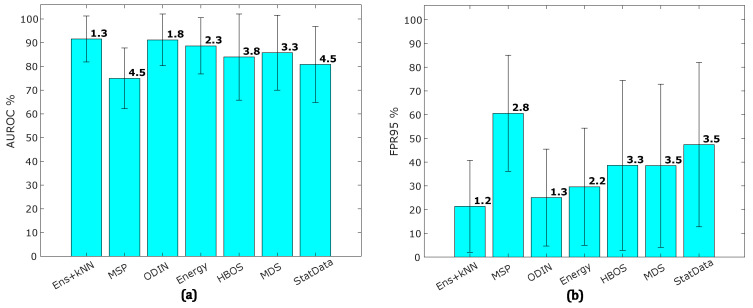
The results of the OOD detection methods after applying kNN to the OOD scores from individual models: (**a**) the average AUROC across all methods and (**b**) the average FPR95 for each method. Error bars represent standard deviations across datasets.

Table 3 and Figure 6 show a large gap between the performance of OOD detection from single models and ensemble models. To explore a combined OOD detection approach, we aggregated the OOD detection outputs of the base models as features. Specifically, kNN was applied to aggregate features from MSP, ODIN, Energy, HBOS, and MDS methods for comparison. Figure 7 illustrates the average AUROC and FPR95 achieved by each OOD detection method when using kNN with k=1 to aggregate features from the ensemble models. While Ens+kNN continues to achieve the highest average AUROC and lowest FPR95, other methods also show notable improvement. Conversely, StatData now exhibits the second lowest performance, with MSP achieving the lowest performance.

## 6. Discussion

The multi-sensor deep ensemble workflow performed remarkably well across datasets with a wide range of properties, benefiting from automatic HPO. The dataset parameters included sensor counts from 3 to 17 and signal lengths ranging from 60 to 13,108. For all datasets, the ID test accuracy was close to 100%, indicating the model’s ability to generalize within the provided training set.

The number of models in the final ensemble, denoted as *m*, is an HP that directly influences prediction outcomes. Previous research, such as [28], suggests that increasing *m* enhances prediction performance, but this comes with the trade-off of increased ensemble size and computational demands. It is important to strike a balance between the benefits of more models and the practicality of implementation. Moreover, while ensemble size also affects OOD detection performance, our model selection process was conducted independently of the OOD samples, focusing primarily on ID performance.

In this study, we treated each possible shift in the data distribution as a true label for OOD detection. While the true labels of distribution shifts were not available and this assumption may not perfectly represent the problem, the results effectively highlight the existence of the issue and demonstrate the method’s potential in identifying shifts. Developing specialized datasets targeting distribution shifts in this field would be valuable for future research [70].

Figure 4b illustrates significant variation in model accuracy on OOD data across different domains, validating the presence of the problem in the defined scenarios. These variations differ between datasets, indicating that the severity of distribution shifts depends on the covariate variables. For example, in the HS (Valve) dataset, OOD accuracy ranged from 33% to 85%, with the lower value approximating random guessing. This variability highlights the complexity of OOD detection, as some distributional shifts pose substantial challenges to the model, while others have minimal impact on prediction results. The selection of k=1 for kNN-based OOD detection was motivated by maximizing domain shift sensitivity, but this choice might not be optimal for all datasets. Larger *k* values could smooth out the influence of noise and improve OOD detection in certain contexts, warranting further exploration of dataset-specific HPO.

Despite the small gap between the ID prediction accuracy of single models and the multi-sensor deep ensemble, the OOD detection performance of single models was notably inferior. As shown in Figure 6, OOD detection methods based on single models consistently exhibit high FPR values, averaging around 90%. However, the presence of domain shift is evident, as reflected by the severe drop in OOD prediction accuracies, with the EA dataset dropping to around 10% and the HS (Acm) dataset ranging between 3% and 27%. Conversely, Ens+kNN achieved near-perfect prediction metrics. This inconsistency suggests that focusing solely on ID accuracy for model selection in multi-sensor datasets is insufficient. Additional factors must be considered to ensure robust model performance.

Aggregating information from input signals across multiple sensors, “StatData” has shown potential for detecting shifts in data distribution early on. When compared to single-model OOD detection methods, StatData ranked as the second-best approach, delivering strong AUROC results, particularly for the EA and HS datasets, including both the Valve and Acm tasks. However, the method exhibited significant variability in FPR95, ranging from 10% to 80%, underscoring its inconsistency in detecting certain types of domain shift. Compared to other aggregation-based methods, StatData had the second-highest FPR95, making it less reliable for scenarios requiring low false positive rates. Nonetheless, since it operates independently of the model architecture, StatData can serve as a pre-model selection tool, highlighting potential shifts in data before more computationally intensive models are deployed.

## 7. Conclusions

In this study, we proposed a robust framework tailored for multi-sensor applications, particularly valuable in high-stakes and costly decision-making areas such as industrial fault detection systems. Our approach demonstrated leading-edge performance across various datasets, accommodating diverse signal lengths and sensor counts. Its adaptability and reliability across the datasets highlight its promising generalizability. While a collection of widely used datasets was employed in this study, the generalizability of the framework to other industrial scenarios may depend on factors such as task specifications, sensor types, and the nature of domain shifts encountered. Future work should explore these aspects to better understand the framework’s potential and limitations in broader industrial applications.

The datasets used in this study are not explicitly designed to capture domain or distribution shifts. We treated any deviations from the initial working conditions as potential shifts in the distribution. However, further investigation is needed to quantify the degree of deviation from the training distribution within these datasets. Additionally, developing datasets specifically designed to address domain shifts in this field would be highly valuable.

The framework’s model-agnostic design allows it to be extended to ML models beyond DNNs. Although the models in this study were limited to ConvNets, exploring a broader range of DNN architectures or a combination of different models could enhance ensemble diversity, offering a promising direction for future research. Additionally, future studies could investigate different fusion methods and assess how the choice of fusion depth (defined as the stage at which features or decisions are integrated) influences overall performance.

This study’s unique contribution lies in its AutoML approach, which addresses diverse datasets from multi-sensor fault detection use cases while accounting for distribution shifts. By leveraging a flexible design, the framework effectively accommodates variations in sensor data, ensuring consistent performance across a wide range of datasets. While the findings are promising, we hope this work inspires further research to refine and expand similar approaches, enhancing the practical applicability of ML methods in critical industrial settings.

## Figures and Tables

**Table 1:** Design choices for each block in the proposed framework.

Framework Block	Selected Design Choice
HP-Driven Ensemble	Deep Ensemble using ConvNets
Fusion	Concatenation of softmax scores
Meta-Classifier	AdaBoost
Anomaly Detection	kNN

**Table 2:** Overview of the datasets, including signal lengths, the number of classes in the defined classification task, the number of domains, and the number of sensors.

Dataset	Signal Length	Num. Classes	Num. Domains	Num. Sensors
EA	[2000]	5	4	11
HS *	[60, 600, 6000]	4	3	17
OGW	[256, 6554, 13,108]	2	4	3
PU	[2048, 4096]	2	4	4
CWRU	[512, 1024]	4	4	4

* Two classification tasks are generated from the HS dataset, detecting fault type of the accumulator (Acm) and valve (Valve).

**Table 3:** Performance comparison across six datasets and seven methods. The arrows (↓ and ↑) next to the metrics indicate whether lower or higher values are better, respectively.

Method	EA		HS (Acm)		HS (Valve)		OGW		PU		CWRU	
	AUROC ↑	FPR ↓	AUROC ↑	FPR ↓	AUROC ↑	FPR ↓	AUROC ↑	FPR ↓	AUROC ↑	FPR ↓	AUROC ↑	FPR ↓
Ens+KNN	100.0	0.0	100.0	0.0	100.0	0.0	75.7	61.7	92.0	21.7	81.8	45.1
MSP	82.9	62.2	94.5	99.2	99.3	100.0	55.3	79.3	50.4	88.5	52.2	89.5
ODIN	99.2	100.0	100.0	100.0	67.8	80.3	52.2	88.5	52.1	89.5	81.4	64.5
Energy	97.2	99.9	100.0	100.0	70.0	83.6	51.5	89.5	51.1	89.3	77.0	73.1
HBOS	98.1	99.8	94.5	100.0	68.2	97.9	55.9	100.0	50.3	89.0	64.3	81.7
MDS	89.8	99.9	77.3	93.0	75.6	100.0	56.1	97.1	52.6	95.4	71.2	62.1
StatData	82.3	33.3	99.4	0.0	100.0	0.0	54.8	89.6	69.1	99.7	79.2	61.8

**Figure A1:**
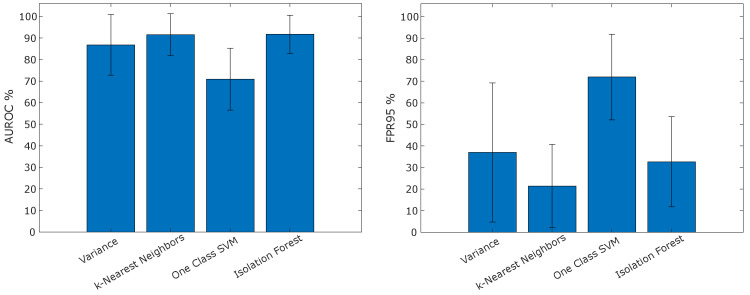
Average AUROC and FPR95 values for each anomaly detection method across all datasets.

## Data Availability

All datasets used in this study are publicly available. Detailed information on the sources of these datasets can be found in the corresponding sections of this paper.

## Abbreviations

    The following abbreviations are used in this manuscript:

AUROC	Area Under the Receiver-Operating Characteristic Curve
OOD	Out-of-Distribution
ID	In-Distribution
TPR	True Positive Rate
FPR	False Positive Rate
FPR95	FPR at 95% TPR
kNN	k-Nearest Neighbors
MSP	Maximum Softmax Probability
MDS	Mahalanobis Distance-Based OOD Detection
HBOS	Histogram-Based Outlier Score
ConvNet	Convolutional Neural Network
HP	Hyperparameter
EA	The ZeMA Electromechanical Axis Dataset
HS	The ZeMA Hydraulic System Dataset
OGW	The Open Guided Waves Dataset
PU	Paderborn University Bearing Dataset
CWRU	Case Western Reserve University Bearings Dataset
ML	Machine Learning
AutoML	Automated Machine Learning
DNN	Deep Neural Network
StatData	OOD Detection Using Statistical Moments of Data
HPO	Hyperparameter Optimization

## Appendix A. Datasets

### Appendix A.1. ZeMA Electromechanical Axis (EA) Dataset

The EA dataset [65] was recorded during the lifetime assessment of electromechanical axes following a consistent working cycle of 2.8 s, including a forward stroke, idle time, and a return stroke. The dataset comprises one second of the return stroke from every 100th cycle, making it suitable for evaluating remaining useful life.

**Classes:** The target variable consists of five categories, from ‘1’ (new device) to ‘5’ (nearing failure).

**Sensors:** Data was recorded from 11 sensors, each with a sampling rate of 2 kHz.

**Sequence lengths:** Each sensor provides 2000 samples per observation.

**Domains:** Data from four distinct devices is included, with each device representing a separate domain to assess model generalizability.

### Appendix A.2. ZeMA Hydraulic System (HS) Dataset

The HS dataset [66] contains recordings from a testbed designed to simulate various fault conditions of a real hydraulic system. The target variables are the accumulator (Acm) pre-charge pressure and valve conditions. For this study, the dataset is split into two subsets: HS (Valve) and HS (Acm).

**Classes:** The goal is to assess the fault severity in each task. Both versions define four fault categories as target classes.

**Sensors:** The testbed includes 17 sensors: six pressure sensors (100 Hz), four temperature sensors (1 Hz), two volume flow sensors (10 Hz), motor power (100 Hz), and one vibration sensor (1 Hz). Additionally, three virtual sensors with a 1 Hz sampling rate are included for cooling efficiency, cooling power, and efficiency factor.

**Sequence Lengths:** Each observation consists of 6000, 600, or 60 data points, depending on the sampling rate.

**Domains:** Cooler performance levels at 3%, 20%, and 100% are considered distinct domains, with 100% assumed as the source domain.

### Appendix A.3. Open Guided Waves (OGW) Dataset

The OGW dataset [67] contains time-series signals capturing guided waves at various temperatures, ranging from 20 °C to 60 °C in increments of 0.5 °C. Signals were recorded using ultrasonic transducers arranged in a sender-receiver configuration, attached to a carbon fiber reinforced polymer (CFRP) plate. To simulate delamination damage, a detachable aluminum mass was placed at four different locations on the plate. Baseline measurements were collected on an intact CFRP plate at the same temperature levels.

**Classes:** This dataset is used for binary classification to identify whether the CFRP plate is damaged or intact.

**Sensors:** The preprocessed version of the recorded signal serves as the primary sensor. A single-sided FFT amplitude of this signal provides a virtual sensor. Additionally, due to higher power in the low-frequency spectrum, the first 256 data points from the FFT sensor are used as a third sensor.

**Sequence Lengths:** The primary sensor provides 13,108 data points per observation, the second sensor 6554, and the third sensor 256.

**Domains:** Measurements were conducted with simulated damage at each of four positions on the plate, which serve as different domains. The source domain is defined as the simulated fault at position $D_{12}$.

### Appendix A.4. Paderborn University (PU) Bearing Dataset

The PU dataset [68] includes recordings of high-frequency vibrations and motor currents from 32 bearings, comprising 26 faulty bearings and 6 healthy bearings.

**Classes:** This dataset is used for binary classification, aiming to distinguish between faulty and healthy states.

**Sensors:** The primary sensors used are vibration and current signals, both sampled at 64 kHz. The original four-second signals are segmented into non-overlapping slices with 4096 data points. To incorporate frequency domain data, a single-sided FFT was applied to the vibration signals, providing a virtual sensor. This study utilizes four sensors: vibration, current, FFT-transformed vibration data, and additional measurements (speed, load, torque, and temperature) to capture working conditions.

**Sequence Lengths:** Three sensors provide 4096 data points per observation, while one provides 2048.

**Domains:** The data was collected under four different working conditions, each representing a distinct operating scenario. In this study, the working condition with a rotational speed of 900 RPM, a torque load of 0.7 Nm, and a radial force of 1000 N is designated as the source domain.

### Appendix A.5. Case Western Reserve University (CWRU) Bearing Dataset

The CWRU dataset [69] is widely used in the predictive maintenance domain. It consists of vibration signals from bearings under different fault conditions and varying load settings. The dataset includes recordings from “healthy”, “inner ring fault”, “outer ring fault”, and “ball fault” states.

**Classes:** The task is a binary classification, aiming to differentiate between faulty and healthy states.

**Sensors:** Physical sensors include vibration signals from the drive end and fan end. Additionally, the single-sided FFT amplitude of each signal serves as two virtual sensors, resulting in a total of four sensors.

**Sequence lengths:** Data is sampled at 12 kHz and segmented into non-overlapping 1024-sample slices for each observation. Each virtual sensor provides 512 samples per observation.

**Domains:** Recordings are taken under different motor loads (0, 1, 2, and 3 HP), with each load representing a separate domain. The 1 HP load is the source domain, with its recordings designated as ID samples.

## Appendix B. HyperParam Convolutional Neural Network

HP-ConvNet is a Convolutional Neural Network (ConvNet) designed with tunable HPs to optimize its performance. The primary component of the ConvNet is the convolutional layer, for which three key HPs are considered:

1. kernel size, which defines the size of each filter used in the network to capture features;

2. stride size, which specifies the step size by which the filter moves across the input;

3. the number of kernels, which indicates the number of filters used in each layer, controlling the network’s capacity to extract features.

All convolutional layers in the network share the same kernel size, except for the first convolutional layer. Following the approach in [71], the first convolutional layer can have a wider kernel size and a larger stride, referred to as the First Kernel Size and First Stride Size.

The number of kernels in each convolutional layer is determined as a function of the previous layer’s kernel count and a Kernel Growth Factor, as described in Algorithm A1. The kernel count increases by multiplying the previous layer’s kernel count by the growth factor after every Kernel Growth Period. For layers outside the growth period, the kernel count remains the same as the last updated value.

Convolutional layers can reduce the feature size by using stride sizes greater than one. The first convolutional layer uses a separate stride size. Starting from the second convolutional layer, the stride size is either the defined stride size or one, depending on a HP called the Stride Period, which controls when the stride size exceeds one. Algorithm A2 explains computing the stride sizes of the convolutional layers.

**Algorithm A1** Determining the Number of Kernels in Each Layer
1: **Initialize** kernel_counts 2: current_kernels←initial_kernels 3: **for** layer_index←1 **to** num_layers **do** 4: **if** layer_index==1 **then** 5: Append current_kernels to kernel_counts 6: **else** 7: **if** (layer_index)modkernel_growth_period==0 **then** 8: current_kernels←⌈current_kernels×growth_factor⌉ 9: **end if** 10: Append current_kernels to kernel_counts 11: **end if** 12: **end for** 13: **return kernel_counts**

**Algorithm A2** Determining the Stride Size in Each Layer
1: **Initialize** stride_sizes 2: **for** layer_index←1 **to** num_layers **do** 3: **if** layer_index==1 **then** 4: Append first_stride to stride_sizes 5: **else** 6: **if** (layer_index−1)modstride_period==0 **then** 7: Append stride to stride_sizes 8: **else** 9: Append 1 to stride_sizes 10: **end if** 11: **end if** 12: **end for** 13: **return stride_sizes**

Dropout [72] is a common regularization method used in deep neural network (DNN) architectures to prevent overfitting. The dropout rate specifies the percentage of neurons to be deactivated during each training iteration. In HP-ConvNet, dropout rate is a tunable HP. Each convolutional block, except the first one, can include a dropout layer. The inclusion of dropout layers begins at a specified position, determined by the HP StartDropout. Starting from the *k*-th convolutional block (defined by StartDropout), all subsequent blocks incorporate a dropout layer.

Global pooling layer is effective to reduce the number of features after feature extraction by the convolutional blocks. HP-ConvNet has a Global pooling layer or alternatively a fully connected layer. If there is a fully connected layer, Num. Neurons defines the number of neurons in the layer.

Table A1 summarizes the search space for the Ps of the HP-ConvNet during Bayesian optimization. The primary focus of the HPO is on architectural HPs rather than training-related HPs.

**Table A1:** Automated Hyperparameter Search Space for ConvNets. The HPs are divided into two groups: architectural HPs and training HPs.

Architectural HP	Values	Training HP	Values
Initial Num. Kernels	[10, 100]	Max Epochs	200
Num. Neurons	[200, 800]	LR Drop Factor	0.95
Num. Conv Blocks	[3, 10]	Validation Patience	10
First Kernel Size	[7, 50]	Mini Batch Size	128
First Stride Size	[1, 50]	Initial LR	[0.001, 0.01]
Kernel Size	[3, 9]	Optimizer	Adam
Kernel Growth Period	[1, 4]		
Kernel Growth Factor	[1, 2]		
Stride Size	[1, 9]		
Stride Period	[1, 4]		
Global Pooling	[false, true]		
Dropout Rate	[0.2, 0.6]		
Start Dropout	[2, 5]		

## Appendix C. Hyperparameter Optimization Constraints

### Appendix C.1. Convolutional Layers

Kernel size and stride are the primary properties of a convolutional layer. These parameters influence how input data is processed and the resulting feature map size. A constraint must be satisfied in each convolutional layer:

(A1) Stride≤KernelSize

This ensures sufficient coverage of the input data and avoids situations where the stride size exceeds the kernel size, which could result in skipped input regions.

### Appendix C.2. Learnable Parameters

Learnable parameters in a ConvNet are primarily composed of kernel weights in the convolutional layers and weights in the fully connected layers. Excluding bias terms, the number of learnable parameters in each convolutional layer can be calculated as follows:

(A2) LearnableParameters=(KernelHeight×KernelWidth×InputKernelCount)×OutputKernelCount

Minimizing the number of learnable parameters is advantageous for various reasons. Smaller networks require fewer training resources, such as computational power and memory, and consume fewer resources during inference after deployment. To address this, we impose a constraint on the number of learnable parameters:

(A3) LearnableParameters<MaxLearnableParameters

In this study, Max Learnable Parameters is defined based on hardware constraints and is limited to 200 million learnable parameters.

### Appendix C.3. Receptive Field

The receptive field of a convolutional network determines the size of the input region that influences a single output feature. The receptive field should be sufficiently large to capture meaningful patterns but not excessively large to remain practical for a given task. To ensure this, the following constraint must be satisfied:

(A4) ReceptiveField≤MaxReceptiveFieldSize.

A practical heuristic for defining this constraint is to base the maximum receptive field size on the length of the input signal. This approach avoids unnecessarily large or ineffective receptive fields. In this study, the receptive field is limited to 1.5 times the length of the input signal to achieve an optimal trade-off between efficiency and effectiveness.

## Appendix D. Anomaly Detection Techniques for OOD Scoring

Deriving the OOD score from the aggregated scores can be achieved using various anomaly detection methods. In this study, we applied four different approaches to compute the OOD scores. These methods range from simple statistical measures to advanced ML techniques.

**Variance**: Without employing additional models, the simple variance of outputs across different models can serve as an indicator of uncertainty in the final prediction. Higher variance often correlates with greater uncertainty, suggesting potential OOD instances [28].

**k-Nearest Neighbors** (kNN): The kNN algorithm identifies the OOD score by computing the distance of a given sample to its nearest neighbors in the feature space. Samples with larger distances to the closest ID points are more likely to be classified as anomalies [43].

**One-Class Support Vector Machine** (OC-SVM): This method maps the data into a high-dimensional feature space and identifies a hyperplane that separates the ID data from potential anomalies [73].

**Isolation Forest**: The algorithm builds decision trees to partition the data and identifies anomalies as points that require fewer splits to isolate [74].

Figure A1 presents the average AUROC and FPR95 values for all anomaly detection methods evaluated across six datasets.
